# Case of an Extra-Uterine Fœtus

**Published:** 1787

**Authors:** Edward Jacob

**Affiliations:** Member of the Corporation of Surgeons of London, and Surgeon at Faversham, in Kent


### VIII. *Caſe of an Extra-uterine Fœtus. Communicated in a Letter to Dr.* Simmons, *by Mr.* Edward Jacob, *junior, Member of the Corporation of Surgeons of London, and Surgeon at Faverſham, in Kent.*

MARY Jacob, a poor woman, aged twenty-three years, of a middle ſtature, and of a thin but healthy habit, became pregnant in the beginning of the year 1785. In a former pregnancy ſhe had miſcarried about the ſixth month, in conſequence of a fright, and the fœtus had been ſuddenly brought into the world by the natural pains; but the placenta having adhered, and a flooding followed, I had been called to her aſſiſtance, and ſoon extracted it. From that time ſhe had recovered, and remained in perfect health. In this ſecond pregnancy no particular ſenſations led her to ſuſpect that ſhe had conceived, till an obſtruction of the menſes took place; after which ſhe ſuffered more ſeverely than common that train of ſymptoms ſo often attendant on early pregnancy, ſuch as conſtant colic, ſickneſs, coſtiveneſs, dyſury, and want of natural reſt; but without ſeeking any aſſiſtance from medicine.

In the beginning of June ſhe firſt felt the motion of the child. This motion ſhe deſcribed as a kind of plunging within her, attended with ſevere pain, and which continued almoſt without ceſſation, and was ſo violent, that at the end of a fortnight convulſive fits were the conſequence, and I was again applied to. Pain appearing to have been the immediate cauſe of theſe convulſions, I placed my chief reliance on opiates; but although ſtrong ones were adminiſtered, they were too weak to remove the pain; yet, though her ſituation was ſuch, no other marks of miſcarriage ever preſented themſelves.

As the patient was ſeveral miles diſtant from me, I neither ſaw nor heard of her from June 23d till Auguſt 15th, when a meſſage being ſent to me, deſcribing her to have continued nearly in the ſame diſtreſsful ſituation as before, an opiate and clyfter were directed to be adminiftered. From that time I received no farther account of her till October 17th, when I was called to attend her in her fuppofed labour, her friends judging, from her melancholy fituation, that, unlefs fhe was then delivered, life could not long be fupported. On my entering her chamber, I was ftruck with her melancholy appearance, as I found her with a deadly countenance, and emaciated to the greateft degree at every part, except the abdomen, which was as much on the ftretch as it commonly is in the laft week of pregnancy.

The account fhe then gave me of herfelf was, that in the month of Auguft, after having had recourfe to the opiate and clyfter I had recommended to her, fhe had dreamt that fhe was falling from fome precipice, and that having waked fuddenly in a great fright, fhe had found an alteration in the fituation of the child within her; for it now feemed to her as though it lay quite round the infide of the abdomen, and from that inftant it never moved but as a heavy, dead weight; that afterwards fhe could never lie down in bed, was never free from pain, and could in no refpect attend attend to her family affairs; and yet the abdomen did not in the leaſt decreaſe in ſize.

On inquiry, I found that her pain was conſtant, and yet not like forcing labour pain, and that ſhe had no other marks of labour; for on examining the os tincæ, it felt rigid and cloſe ſhut; the neck of the uterus ſeemed to be of its natural length, and there was no perceptible increaſe of bulk in the body of it: yet, with all theſe circumſtances, a child was plainly to be felt through the parietes of the abdomen.

I now again had recourſe to an opiate, with the hope of its leſſening the ſeverity of her pain, and the next day I had the pleaſure to learn that eaſe and ſleep had been obtained by it; I therefore ordered it to be repeated every night, and at any time when occaſion might require.

From this time her bulk began gradually to decreaſe, though a circumſcribed hardneſs was always to be felt around the umbilicus. I occaſionally after this viſited her, and deſired her to be particular in her remarks, that I might be informed of every change that might take place in her.

No

No difcharge of any kind had ever appeared, *per vaginam,* from the firft obftruction of the menfes till February, 1786, when they appeared again, and continued to return regularly till Auguft, when they flowed in greater quantity than ufual, but did not return afterwards. An inflammation, which had been for fome time more or lefs round the umbilicus, now terminated in fuppuration, and a fœtid matter began to flow therefrom, gradually increafing in quantity. Soon after this change I faw her, and fhe appeared to have no other complaints than what fuch an inconvenience muft occafion, her pain not being very diftreffing.

After this I heard no more of this poor woman till January, 1787, when Mr. Curteis, Surgeon of this place, (to whom I had from the firft mentioned the nature of her cafe, and her name) informed me, that the overfeers of her parifh had defired him to attend her. On his firft vifit to her he had difcovered fome bones of a fœtus prefenting through the opening at the umbilicus, whence the fœtid matter had fo long been difcharged; and he was afterwards fo obliging as to give me an opportunity of attending her with him.

On

On our examining her together, the ſpine and back part of the ribs of the fœtus were found preſenting at the opening, which at this time was nearly of the breadth of half a crown, and through which two of the ſpinal vertebræ were eaſily ſeparated. The opening daily became larger, and more of the fœtus diſcovered itſelf. The patient was kept as clean as her ſituation would permit; but notwithſtanding every care, the putrid ſtench was ſo great, that her room was almoſt intolerable. In this deplorable ſituation opiates were freely exhibited, and the ſtricteſt attention was paid to the non-naturals. At our third viſit appearances were more favourable, the nates of the child preſenting through the opening ſo far as to enable me to wrap a cloth round them, and to bring them forward. The parts, from ſo long a putrefaction, being looſened, with little pain yielded to my endeavours, and the whole of the fœtus ſeemed to be brought away at once, by a ſteady perſeverance, without any dilatation or great force; but upon examining the opening, one of the parietal bones was found ſeparated, which, with part of one of the legs, was brought away by the common forceps; but the former bone, from its ſaw-formed edge being being detached from the head, gave more of pain than all the reſt of the child, and yet not a ſingle blood veſſel was broken in the attempt. After every extraneous matter was removed, we applied lint only to the part affected, and, by guarding againſt any impediment that might obſtruct nature in her efforts, we had the ſatisfaction to ſee our patient perfectly recovered from every complaint, the parts having contracted and healed in about a month after the child was extracted.

The child, after maceration in water, appeared to be male, was perfect in ſhape, though highly putrid, and meaſured about fourteen inches in length. The funis entered into the umbilicus at its natural place; but not more than three or four inches of it were left.

*Faverſham,*

April 27th, 1787.

